# Two Unique Cases of X-linked SCID: A Diagnostic Challenge in the Era of Newborn Screening

**DOI:** 10.3389/fped.2019.00055

**Published:** 2019-04-05

**Authors:** Pooja Purswani, Cristina Adelia Meehan, Hye Sun Kuehn, Yenhui Chang, Joseph F. Dasso, Anna K. Meyer, Boglarka Ujhazi, Krisztian Csomos, David Lindsay, Taylor Alberdi, Sonia Joychan, Jessica Trotter, Carla Duff, Maryssa Ellison, Jack Bleesing, Attila Kumanovics, Anne M. Comeau, Jaime E. Hale, Luigi D. Notarangelo, Troy R. Torgersen, Hans D. Ochs, Panida Sriaroon, Benjamin Oshrine, Aleksandra Petrovic, Sergio D. Rosenzweig, Jennifer W. Leiding, Jolan E. Walter

**Affiliations:** ^1^Johns Hopkins All Children's Hospital, St. Petersburg, FL, United States; ^2^Division of Allergy and Immunology, Department of Pediatrics, Morsani College of Medicine, University of South Florida, Tampa, FL, United States; ^3^Immunology Service, Department of Laboratory Medicine, Clinical Center, National Institutes of Health, Bethesda, MD, United States; ^4^Department of Biology, University of Tampa, Tampa, FL, United States; ^5^Division of Allergy and Immunology, Department of Pediatrics, University of Texas Medical Branch, Galveston, TX, United States; ^6^Dearborn Allergy & Asthma Clinic, PC, Dearborn, MI, United States; ^7^Cincinnati Children's Hospital Medical Center, Cincinnati, OH, United States; ^8^Department of Pathology, University of Utah, Salt Lake City, UT, United States; ^9^ARUP Laboratories, Institute for Clinical and Experimental Pathology, Salt Lake City, UT, United States; ^10^New England Newborn Screening Program and Department of Pediatrics, University of Massachusetts Medical School, Worcester, MA, United States; ^11^New England Newborn Screening Program, University of Massachusetts Medical School, Worcester, MA, United States; ^12^National Institute of Allergy and Infectious Disease, National Institutes of Health, Bethesda, MD, United States; ^13^Department of Pediatrics, University of Washington & Seattle Children's Research Institute, Seattle, WA, United States; ^14^Division of Allergy and Immunology, Massachusetts General Hospital for Children, Boston, MA, United States

**Keywords:** interleukin 2 receptor gamma (*IL2RG)*, X-linked severe combined immunodeficiency (SCID), newborn screening, maternal X-inactivation studies, functional assays, gamma chain signaling

## Abstract

In the era of newborn screening (NBS) for severe combined immunodeficiency (SCID) and the possibility of gene therapy (GT), it is important to link SCID phenotype to the underlying genetic disease. In western countries, X-linked interleukin 2 receptor gamma chain (IL2RG) and adenosine deaminase (ADA) deficiency SCID are two of the most common types of SCID and can be treated by GT. As a challenge, both *IL2RG* and *ADA* genes are highly polymorphic and a gene–based diagnosis may be difficult if the variant is of unknown significance or if it is located in non-coding areas of the genes that are not routinely evaluated with exon-based genetic testing (e.g., introns, promoters, and the 5′and 3′ untranslated regions). Therefore, it is important to extend evaluation to non-coding areas of a SCID gene if the exon-based sequencing is inconclusive and there is strong suspicion that a variant in that gene is the cause for disease. Functional studies are often required in these cases to confirm a pathogenic variant. We present here two unique examples of X-linked SCID with variable immune phenotypes, where IL2R gamma chain expression was detected and no pathogenic variant was identified on initial genetic testing. Pathogenic *IL2RG* variants were subsequently confirmed by functional assay of gamma chain signaling and maternal X-inactivation studies. We propose that such tests can facilitate confirmation of suspected cases of X-linked SCID in newborns when initial genetic testing is inconclusive. Early identification of pathogenic *IL2RG* variants is especially important to ensure eligibility for gene therapy.

## Introduction

SCID is caused by a defect in cellular and humoral immunity, primarily stemming from abnormal T cell development and/or function. Severe T cell dysfunction will impede effective humoral immunity as B cell responses to most antigens are T cell dependent. Due to the severity of the disease, early diagnosis is essential. After 10 years of implementation process, as of December 2018, all the states in United States (US), District of Columbia and Puerto Rico have implemented newborn screening (NBS) for SCID, thus covering all children born in the United States (1, 2).

When detected by NBS, neonates with typical SCID present asymptomatically due to protection from maternal antibodies and lack of exposure to pathogens, but nevertheless require prompt preparation for allogeneic or autologous (gene therapy) hematopoietic stem cell transplantation (HSCT) to prevent fatal outcomes from infections. With advances in genetic sequencing technology, pathogenic variants have been discovered in over 20 genes that cause classic or leaky SCID phenotypes (3, 4). Novel genes contributing to a SCID phenotype continue to be discovered such as the recent finding of a frameshift variant in the linker for activation of T cells (*LAT*) (4), moesin (*MSN*) (5), and *BCL11B* genes (6, 7). The most common genetic cause of SCID in the developed world are variants in *IL2RG*, which encodes for the common gamma chain (γc) of the interleukin-2 (IL-2) receptor. The common γc is also shared by leukocyte receptors for other cytokines (IL-4, IL-7, IL-9, IL-15, and IL-21) that are relevant in T cell, natural killer (NK) cell, and memory B cell development; therefore most patients have a T^−^B^+^NK^−^ SCID phenotype (3, 8). These receptor complexes mediate signal transduction through JAK1 and/or JAK3, and various signal transducer and activator of transcription (STAT) molecules, which induce gene transcription in multiple cell types in response to different cytokines, hormones, and growth factors. Specifically, IL-2Rγ activates STAT5b through its dedicated JAK3 in T cells and IL-21Rγ activates STAT3 in T and B cells and therefore proper γc signaling can be measured by *in vitro* STAT5 and STAT3 phosphorylation. Since the gene is on the X chromosome, practically all cases are males and mothers are often carriers with preferential inactivation of the X chromosome with the pathogenic variant.

In humans, null variants in the *IL2R* gene result in typical X-linked SCID with absent peripheral T and NK cells and dysfunctional B cells due to absent γc signaling (9). In contrast, hypomorphic variants in the *IL2R* gene result in atypical X-linked SCID or combined immunodeficiency with variably reduced numbers and/or function of T, B, and NK cells because of partial impairment in γc signaling (10, 11). Typical X-linked SCID is fatal if not corrected early with HSCT whereas patients with hypomorphic variants of *IL2R* may survive untreated and present with autoimmune complications in addition to infections (12).

Currently most practicing immunologists rely on immune phenotyping (T-B+NK-), DNA sequencing, and/or flow cytometry for γc protein (CD132) expression to assess for γc pathology. The drawback of sequencing is that the *IL2RG* gene is highly polymorphic with pathogenic variants in both coding and non-coding regions (13, 14). Whole exome sequencing (WES) may miss variants in deep intronic regions or in the 3' polyA signal sequence that may affect RNA-splicing or stability as has occurred in published reports (11, 15, 16). Flow cytometry for CD132 protein expression can also be misleading as the abnormal γc protein may be present but dysfunctional.

As a feasible functional assay for γc pathology, a recent publication highlighted the clinical importance of testing IL-21-stimulated B cells for γc signaling in X-linked SCID patients (17). Although γc functional assays are evaluated in multiple research laboratories in the US and across the world, clinically, only one laboratory in the US provides such testing (Immunology Diagnostic Laboratory: A Jeffrey Modell Foundation Diagnostic Center, Seattle Children's Hospital, X-SCID Screen by Flow, pSTAT3/5).

These uncertainties in the diagnostic steps point to the importance of creating an algorithm for early identification of X-linked SCID with high confidence including functional assays in addition to germline DNA sequencing.

## Case Presentation

We report two unique cases of X-linked SCID, in which a disease-causing variant could not be identified on initial genetic testing, but functional assays and additional DNA sequencing led to the discovery and/or confirmation of a pathogenic variant.

### Case A

Patient A is a male infant who had a positive newborn screen for SCID in a US state which routinely performs newborn screening. He was born at 39 weeks gestational age via spontaneous vaginal delivery without prenatal or delivery complications. Around 3 h of life, he developed respiratory distress and required continuous positive pressure ventilation. His newborn screen for SCID with T cell receptor excision circles (TRECs) <20 copies/μL suggested a SCID diagnosis. He was subsequently referred for further immunological evaluation. Absolute lymphocyte counts were low, including near absence of T cells and low NK and B cell counts. Notably, the ratio of CD4+CD45RA+ naïve to total CD4+ T cells was also very low (11%) (Table 1). These findings suggested abnormal thymic T cell maturation.

**Table 1:** Clinical history and immune phenotyping of patients A and B, and additional studies on family members.

		**Patient A (<1 mo)**	**Patient B (9 mo)**	**Reference range**
Clinical presentation at time of lab evaluation		asymptomatic	PJP pneumonia on steroids	
Phenotype of SCID variant		T^−^/B^+^/NK^low^	T^+^(CD8^low^)/B^+^/NK^low^	
TREC (copies/microliter) at birth*		0 (L*)	0 (L*)	>15 [Patient A NBS in Florida], > 252 [Patient B NBS Massachusetts]
Absolute lymphocyte (cells/μL)		669 (L)	1,596 (L)	>2,600
Absolute CD3 T (cells/μL)		9 (L)	1,062 (L)	>1,600
Absolute CD4 T (cells/μL)		9 (L)	1,043	>1,000
Absolute CD8 T (cells/μL)		7 (L)	27 (L)	>400
Absolute CD19 B (cells/μL)		450 (L)	365 (L)	>600
Absolute CD56 NK (cells/μL)		61 (L)	145 (L)	>200
CD4+CD45RA+ naïve T (cells/μL), (% of total CD4)		1 (11%) (L)	0 (L)	>50% of total CD4
CD8+CD45RA+ naïve T (cells/μL), (% of total CD8)		2 (30%) (L)	0 (L)	>50% of total CD8
CD3+ T cell proliferation to mitogens	PHA	0.9% (L)	7.1% (L)	>58.4%
	PWM	11.5%	4.1%	>3.4%
T cell proliferation with interleukins	anti-CD3			
	anti-CD3/CD28	n.a.	Absent	
	anti-CD3/IL-2			
CD132 (common γ chain) expression	T cells	n.a.	84% (L)	>99%
	B cells	87%	84%	>58%
	NK cells	71% (L)	66% (L)	>84%
CD132 signaling		absent	absent	
**X chromosome inactivation (XCI) in carriers**				
Mother	PBMC	76:24	85:15 (H)	<80:20
Mother	T cells	100:0 (H)	100:0 (H)	<80:20
13-year-old sister	PBMC	90:10 (H)	n.a.	<80:20
14-year-old sister	PBMC	91:9 (H)	n.a.	<80:20

X-inactivation studies on peripheral blood mononuclear cells (PBMCs) were performed by Greenwood Genetics (Greenwood, SC). This study does not allow for distinguishing wild type (wt) or mutant alleles. X-inactivation studies in T cells at the NIH allowed for distinction between wt and mutant alleles (Figure 2A). *From NBS card. L indicates low, H indicates high. PJP: Pneumocystis jirovecii pneumonia.

**Figure 1:**
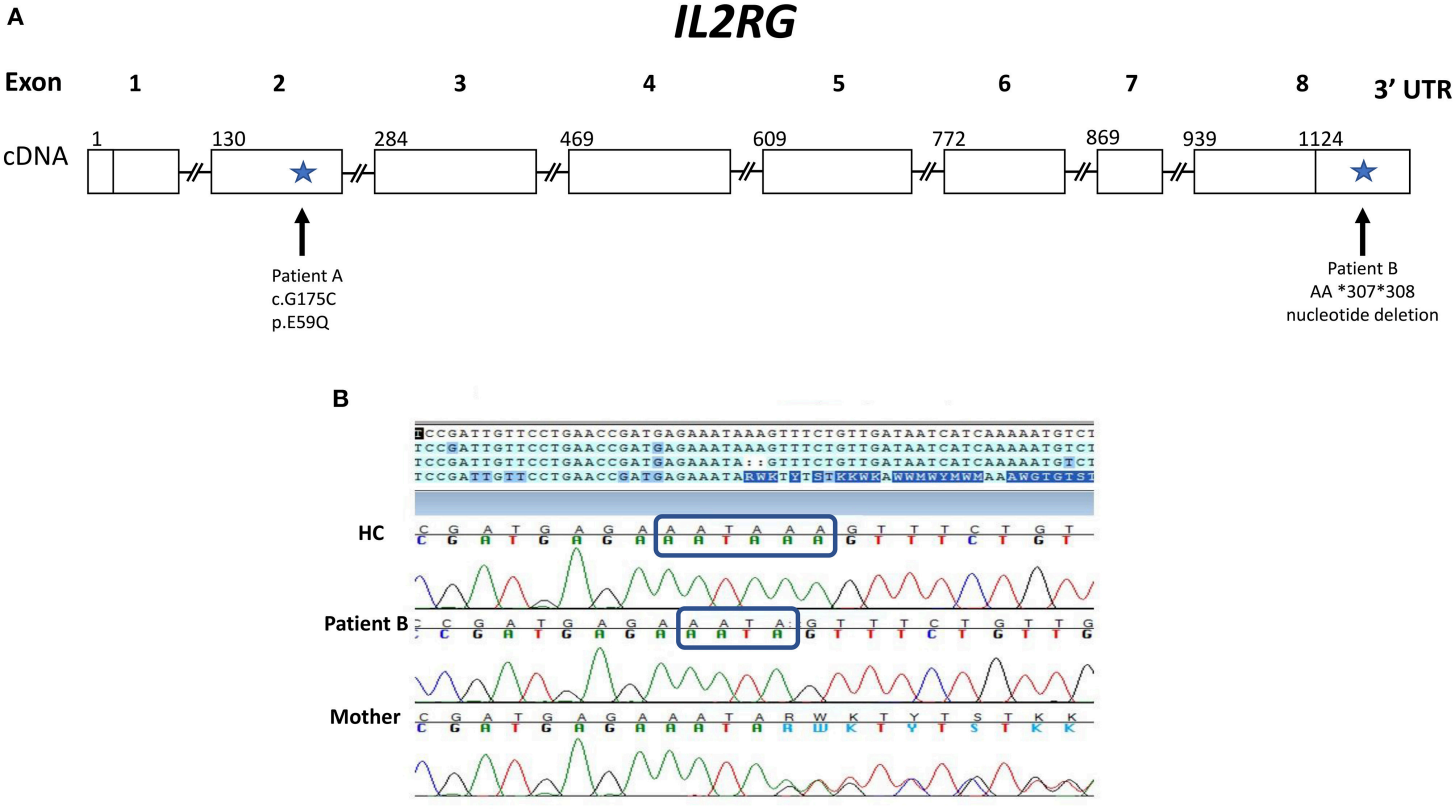
Gene Map of *IL2RG* gene showing novel variants for patients A and B. **(A)** *IL2RG* cDNA map with sites of variants (indicated by arrows) found in patients A and B with X-linked SCID. Patient A has a novel hemizygous missense variant: c.G175C in exon 2 (p.E59Q), which was designated a variant of unknown significance. Patient B has a novel variant in a non-coding region of *IL2RG*: a deletion within the 3′ UTR (AA *307 *308). **(B)** Chromatogram of Sanger sequencing of the 3′ UTR of the *IL2RG* gene for patient B, his mother and a healthy control (HC). Sequencing reveals patient B and his mother (a carrier) have a 2 base pair deletion within the polyA signal sequence: AATAAA is changed to AATA, as indicated by the blue rectangles.

Laboratory evaluation with the carboxyfluorescein succinimidyl ester (CFSE) method revealed decreased CD3+ T cell proliferation with phytohemagglutinin (PHA) (<10% of lower range of normal), but normal B cell proliferation to pokeweed mitogen (PWM) (Table 1). A phenotype of T^−^B^+^NK^low^ SCID was determined based on Primary Immune Deficiency Treatment Consortium (PIDTC) criteria and the patient was referred for evaluation for HSCT (10). In the interim, genetic testing revealed a novel hemizygous missense change in *IL2RG* (c.G175C; p.E59Q), reported as variant of unknown significance (VUS) (Figure 1A). This variant has not been reported in the Exome Aggregation Consortium (http://exac.broadinstitute.org/). His mother and two sisters were carriers of the same variant.

The patient was born to biracial parents and had no matched donor. There was no evidence of maternal engraftment. Therefore, he underwent haploidentical HSCT with his father as donor. He did not receive conditioning and received CD34-selected peripheral stem cell graft. His post-transplant course was complicated by prolonged neutropenia requiring growth factor support and no evidence of T cell reconstitution by 4 months post-transplant. He remained with severe T cell lymphopenia against stem cell boost over 1 year of age. To review his next treatment options including GT, it was necessary to prove that his *IL2RG* variant was pathogenic. Flow cytometry confirmed that the patient had normal level of common γc (CD132) expression on B cells, and low expression on NK cells (Table 1).

Additional tests were initiated to confirm an X-linked inheritance. Under normal circumstances, one of the two X chromosomes is randomly inactivated. Since this is a random process, the ratio of inactivated X_1_ vs. X_2_ chromosomes is less than 80:20. However, if one allele carries a pathogenic variant in *IL2RG*, inactivation is typically highly skewed toward the mutant allele (18). This is especially evident in T cells that are dependent on common γc for their development.

**Figure 2:**
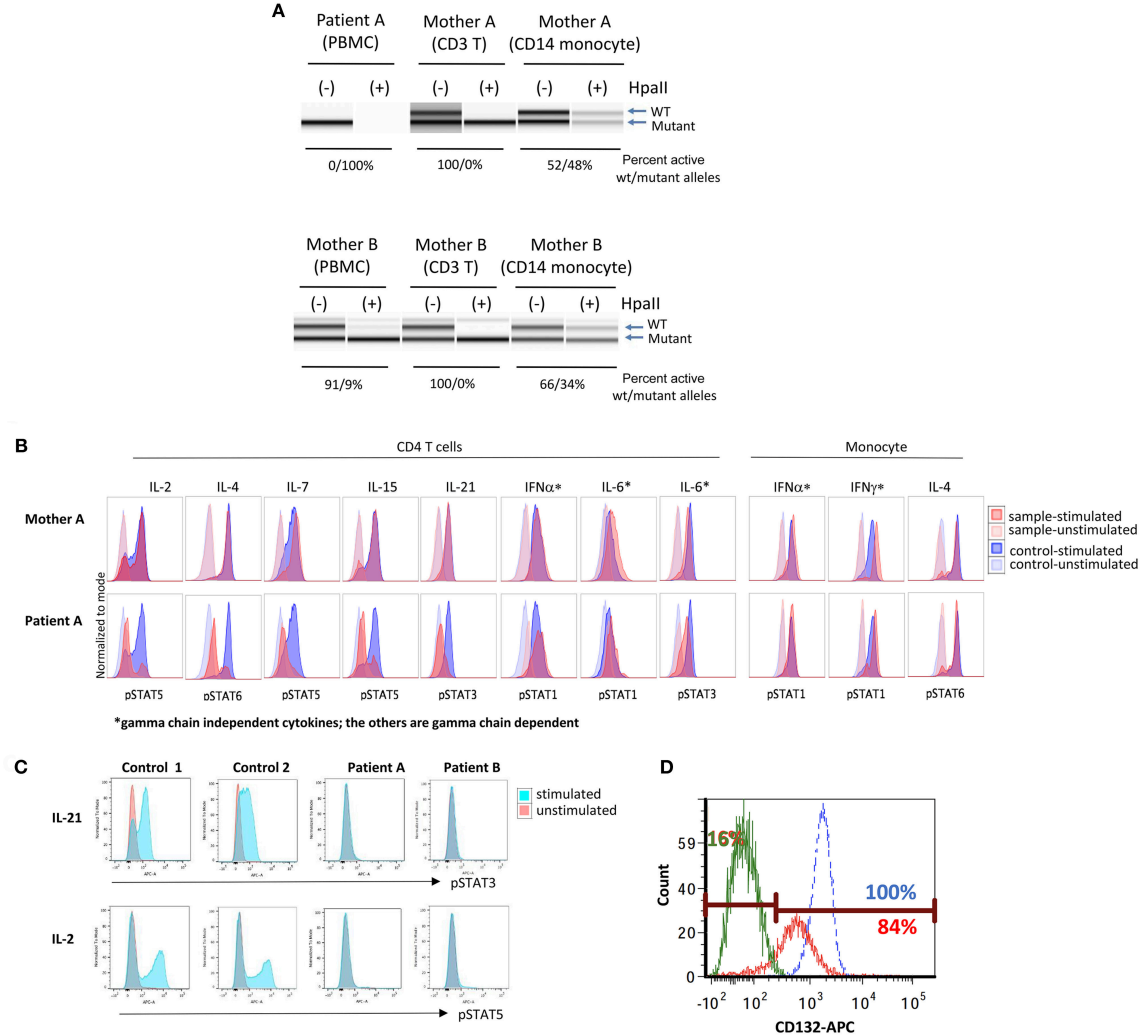
Immune functional assays for interleukin receptor signaling in subsets of lymphocytes. **(A)** X chromosome inactivation (XCI) with (+) or without (−) Hpall. Hpall digests unmethylated alleles. The X chromosome that is active is under methylated, thus, once digested by Hpall, PCR amplification does not occur and band disappears. XCI in CD3+ T cells is skewed toward. **(B)** Histogram of T cell and monocyte response with STAT phosphorylation to several γc dependent (IL-2, IL-4, IL-7, IL-9, IL-15, IL-21) and independent (IL-6, IFN-α, IFN-γ) cytokines in PBMCs of patient A. **(C)** Total lymphocyte response with STAT5 phosphorylation to IL-21 and IL-2 stimulation in patient A, patient B and a healthy control (unstimulated in red, stimulated in blue). **(D)** Level of CD132 (γc) expression on CD3+ T cells of patient B and a healthy control (patient unstimulated in green, patient stimulated in red, and healthy control in blue). Eighty-four percent of patient's cells expressed CD 132 when stimulated, however, mean fluorescence intensity was lower in patient than control. X inactivation and STAT phosphorylation studies in Figures 2A,B were performed by the Department of Laboratory Medicine, Clinical Center, National Institutes of Health (Bethesda, MD).

In genomic DNA (gDNA) extracted from maternal peripheral blood mononuclear cells (PBMCs), X chromosome inactivation (XCI) studies were within normal ranges (76:24). However, XCI studies in maternal T cells did demonstrate complete skewing toward the inactivation of X chromosome with the p.E59Q VUS as verified by two independent laboratories (Table 1 and Figure 2A, upper panel); no preferential inactivation was seen in monocytes, which do not depend on common γc function (Figure 2A, upper panel). Notably, testing of the patient's two sisters (13 and 14 years of age) also showed skewing of X-inactivation in gDNA from PBMCs (90:10 and 91:9, respectively) (Table 1).

Further, signaling studies via common γc in the patient's T cells detected impaired signaling for all γc dependent cytokines (IL-2, IL-4, IL-7, IL-15, and IL-21), while γc independent signaling in T cells (IL-6 and IFN-α) and monocytes (IFN-α and IFN-γ) was preserved (Figure 2B). Subsequent studies for IL-21 and IL-2 signaling in the patient's total lymphocytes demonstrated similar results (Figure 2C). The impaired signaling in γc dependent cytokines and the skewing of X-inactivation in maternal T cells and carrier sisters' PBMCs, taken together, strongly support that the p.E59Q variant detected in *IL2RG* is pathogenic.

### Case B

Patient B presented at 9 months of age with a history of failure to thrive and recurrent upper respiratory infections. He was hospitalized with acute respiratory failure requiring intubation and mechanical ventilation. The diagnosis of *Pneumocystis jirovecii* pneumonia (PJP) was confirmed with bronchoscopy. He was born to non-consanguineous parents under care by a midwife in an out-of-hospital setting in a US state that has fully implemented newborn screening. In keeping with protocol, the original NBS specimen for SCID was rejected for analysis because the sample was insufficient. Repeat testing was delayed due to problems with follow up. However, consented retrieval and retrospective analysis of the original newborn specimen at 9 month of age by an independent and experienced SCID NBS laboratory confirmed the absence of TRECs (Table 1).

During the patient's hospitalization for PJP pneumonia, the absolute lymphocyte count and lymphocyte subsets were mildly decreased. The patient had low CD8+ but normal CD4+ T cell counts (Table 1). However, both CD4+ and CD8+ CD45RA+ naive T cells were absent, indicating impaired T cell development in the thymus. The patient had no evidence of maternal engraftment.

Functional studies were initiated to assess for a SCID phenotype. CD3+ T cell proliferation was poor with mitogen PHA (<30% of lower limit of normal) suggestive of leaky SCID (Table 1) (10). Upon stimulation with anti-CD3 and IL-2, lymphocyte proliferation was absent, which indicated defective T cell receptor signaling. However, γc (CD132) expression was low but detected on T and NK cells, and normal on B cells by flow cytometry (Table 1) (Figure 2D). Mean fluorescence intensity of CD132 was also decreased on CD3+ T cells (Figure 2D). Gamma chain signaling, however, was absent. The diagnosis of ZAP-70 protein deficiency was considered due to low CD8+ and normal CD4+ T cell counts. However, ZAP-70 protein expression was normal by flow cytometry and the gene had no variant.

As a next step, comprehensive genetic testing of 26 genes associated with SCID or combined-immunodeficiency (ADA, AK2, ATM, CD3D, CD3E, CD3Z, CORO1A, DCLRE1C, DOCK8, FOXN1, IL2RG, IL7R, JAK3, LIG4, NHEJ1, ORAI1, PNP, PTPRC, PRKDC, RAC2, RAG1, RAG2, RMRP, STIM1, TBX1, and ZAP-70) was pursued and was unrevealing. Neither did subsequent WES identify a candidate gene.

**Figure 3:**
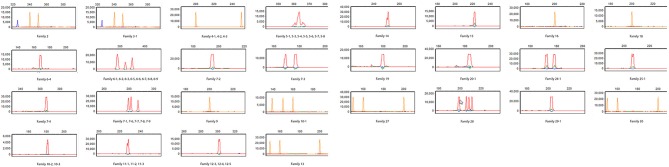
T cell repertoire study. Spectratyping of CD3+ T cells for TCRβ was performed for patient B. The patient has essentially absent TCRVβ repertoire diversity (Cellular and Molecular Immunology Laboratory, Mayo Clinic). The majority of the 23 Vβ families assessed are completely absent and those that show the presence of T cell receptor Vβ families are profoundly oligoclonal (≤5 independent peaks). Patient in red, house-keeping gene (β-actin) in blue, and size marker in orange.

Since immune phenotyping was inconclusive and genetic testing was not informative regarding a causative molecular defect, T-cell repertoire diversity studies with spectratyping of the T-cell receptor beta chain variable region (TCRVβ) at the DNA level were performed. In spectratyping, diversity is estimated by the variation in CDR3 length of mRNA encoding the TCRVβ region. Spectratyping demonstrated that most of the 23 Vβ families assessed were completely absent and the families present were profoundly oligoclonal (≤5 independent peaks) in CD3+ T cells, which was consistent with a T^low^ leaky SCID phenotype (Figure 3).

Maternal XCI studies by two independent laboratories using PBMCs demonstrated mild skewing, while subsequent testing of maternal T cells indicated complete skewing toward inactivation of the X-chromosome with the allele containing the pathogenic variant carried by the patient (Table 1 and Figure 2A lower panel). IL-21 and IL-2 signaling in the patient's total lymphocytes demonstrated impaired signaling (Figure 2C). CD4+ T cell signaling studies could not be done due to inadequate sampling.

The results of the maternal XCI studies and the abnormal IL-2 and IL-21 receptor signaling on isolated lymphocytes suggested an X-linked defect, likely in γc. Further genetic testing of the noncoding regions of *IL2RG*, revealed a 3 prime untranslated region (3′ UTR) deletion NM_000206.2 c.^*^307_^*^308del within the polyA site (AATAAA was changed to AATA) (Figures 1A,B). The patient's mother is a carrier of the same variant. With his mother as donor, the patient underwent haploidentical HSCT and subsequent T and B cell engraftment. The patient was conditioned with fludarabine/melphalan/thiotepa using unmanipulated bone marrow graft from mother. Cyclophosphamide, tacrolimus and mycophenolate mofetil were administered for graft versus host disease prophylaxis. Patient engrafted neutrophils on day19 and platelets on day32 and subsequently demonstrated full T and B cell immune reconstitution.

## Methods

### Measurement of STAT Phosphorylation in Stimulated Lymphocyte Subsets

*IL2RG* γc dependent and independent signaling were studied on gated CD4+ T cells (using APC anti-CD4 from BD Biosciences, La Jolla, CA), monocytes or total lymphocytes using the following cytokines: IL-2, IL-4, IL-6, IL-7, IFN-γ, IL-21 (each 10 ng/mL, except IL-21 50 ng/mL for Figure 2B) (PeproTech, Rocky Hill, NJ), IFN-α (10 ng/mL, Cell Signaling Technology, Danvers, MA), and IL-15 (50 ng/mL, Invitrogen, Waltham, MA). Signaling was assessed using monoclonal antibodies to phosphor-STATs (p-STAT1[Y701], p-STAT3[Y705], p-STAT5[Y694], and p-STAT6[Y641]) (BD Biosciences) and acquired by FACSCanto II flow cytometry. Data was analyzed by FlowJo (Treestar FlowJo, Inc., Ashland, OR).

### X Chromosome Inactivation Assay

Genomic DNA was extracted from the PBMCs of patient and mother using PureLink gDNA minikit (Invitrogen, Waltham, MA). CD3 T and CD14 cells were enriched using EasySep enrichment kit (StemCell Technology, Cambridge, MA). gDNA was first digested with *Rsa*I, then incubated with or without *Hpa*II (Promega, Madison, WI) and finally amplified by polymerase chain reaction using GoTaq DNA polymerase (Promega) (forward primer: 5′-TGCGCGAAGTGATCCAGAACC-3′; reverse primer: 5′-TGGGCTTGGGGAGAACCATC-3′). The products were of different sizes due to the polymorphic nature of the X-chromosome. The percent of active allele was subsequently calculated (19).

### T Cell Diversity Studies

Spectratyping of the TCRVβ family was performed at the Diagnostic Immunology Lab of the Mayo Clinic (Rochester, MN) on clinical grounds using CD3+ T cells from patient B.

## Discussion

Since NBS has been implemented for SCID, the phenotypic and genotypic variability of patients continue to broaden, partly secondary to identification and classification of new hypomorphic variants. We describe two unique cases of X-linked SCID with hypomorphic variants in the *IL2RG* gene. Exon sequencing cannot detect variants located in introns or untranslated regions (patient B), and genetic panel testing may reveal only novel variants of unknown significance (patient A). In either situation, subsequent testing with functional assay of common γc signaling is necessary to confirm whether or not the observed variant is pathogenic, as occurred in the two aforementioned cases.

XCI studies from maternal T cells compared to other hematopoietic lineages are highly useful to support X-linked γc pathology. As T cells are the major, although not only, gDNA contributor to PBMCs, gDNA from other randomly inactivated hematopoietic lineages (e.g., B cells and monocytes) can contribute to falsely normal and misleading results in female SCID-gene carriers. Therefore, when evaluating γc dependent pathology, it is critical to test XCI in separate lineages (e.g., T and NK cells vs. B cells and monocytes) where skewing is differently regulated.

Similar to patient A, there have also been reported cases of atypical X-linked SCID due to a novel variant in the common γc, which required functional analysis to confirm the diagnosis (11, 16). In these two previous cases, the γc protein was truncated or likely truncated whereas in patient A, the protein was detected by flow cytometry (the size of the protein by Western blot was not assessed). Yet in published cases, γc signaling was impaired but not absent, whereas patient A had no γc signaling.

In case of patient B, we report the second known case of a variant in the 3' untranslated region that is associated with X-linked SCID. The first report by Hsu et al. (15) confirmed abnormal mRNA expression due to a single base change from the consensus sequence of the polyA site (AATAAA to AATAAG) (15). We could not accomplish mRNA studies secondary to limited patient sample and inability to establish immortalized cell line from PBMCs despite multiple attempts. It is likely that similar to the previous report, a change from the consensus of AATAAA leads to aberrant transcripts due to the utilization of alternate polyA consensus sequences that causes nonsense mediated decay and unstable transcripts. It is likely that the mRNA is unstable with the polyA tail variant in patient B and this will result in decreased CD132 expression. However, the detection of common γc (CD132) by flow cytometry and impaired signaling through γc indicates a dysfunctional protein. Therefore, it is unclear why signaling is impaired as the γc still could be functional having an intact coding region.

In patient A, the SCID NBS was abnormal and in patient B, NBS was not done at birth but later retrospective studies at about 9 months of age showed absent TREC in a birth specimen. In both cases, the clinical and laboratory findings were consistent with the range of clinical presentation of SCID phenotypes. Common γc expression was detected early and was misleading. Identification of pathogenic *IL2RG* variants associated with abnormal γc signaling and X-linked SCID was delayed for months.

To facilitate identification of γc variants, we propose that in male infants with abnormal SCID NBS that is also confirmed with abnormal lymphocyte subsets, and for whom no pathogenic variant is revealed by DNA sequencing analysis, continue evaluation for *IL2RG* variants using the following studies: (1) screen with X-inactivation studies in maternal T lymphocytes and compare it to other hematopoietic lineages (e.g., B cells or monocytes), and (2) evaluate γc function by assessing its signaling in lymphocytes. These two steps may take only a few days to complete. Even though not all of these tests are commercially available, there is growing evidence to highlight the utility of pursuing functional assays, such as γc signaling in the clinical setting, prior to proceeding with more extensive genetic testing. For instance, whole exome testing takes a few months and is considerably more expensive.

Although SCID patients may receive HSCT before full understanding of their genetic defect, it is important to identify the underlying defect for genetic counseling and targeted GT, especially if a proper donor is not available. In fact, based on a recent consensus approach for the management of SCID caused by ADA deficiency, gamma retrovirus- or lentivirus-mediated autologous HSC-GT is considered an equal alternative (9). This may soon be followed by other gene defects, such as HSC-GT for X-linked SCID. Early identification of a pathogenic variant is critical since minimizing morbidity and mortality of SCID patients is dependent upon timely intervention with GT or allogeneic HSCT.

## Ethics Statement

This study was performed in accord with the recommendations of the ethics committee of Johns Hopkins All Children's Hospital. Patient A and patient B were studied under the Johns Hopkins All Children's Hospital IRB protocol #00097062. The mothers of patient A and patient B gave written informed consent for the publication of the paper.

## Author Contributions

SR and HK from the National Institutes of Health Department of Laboratory Medicine conducted the X-inactivation assay and measurements of STAT phosphorylation in T cells. The laboratory team of TT and HO from Seattle Children's Hospital at the University of Washington conducted the IL-21/IL-2 stimulation studies. Research studies at the University of South Florida and Johns Hopkins All Children's Hospital were conducted by KC, ME, and BU. JH and AC from the University of Massachusetts Medical School evaluated TREC levels from NBS cards for patient B. AK from the University of Utah and YC from Johns Hopkins All Children's Hospital did the genetic testing evaluation and consultation. The laboratory team of JB at Cincinnati Children's Hospital performed the common gamma chain expression studies. The following clinicians, PS, TA, AP, SJ, DL, AM, BO, JL, and JW cared for these patients and were actively involved in research investigations at Johns Hopkins All Children's Hospital. JT, CD, and JL coordinated NBS program. JW, JD, PP, and CM actively wrote the manuscript. LN and JW conceptualized the paper.

### Conflict of Interest Statement

The authors declare that the research was conducted in the absence of any commercial or financial relationships that could be construed as a potential conflict of interest.
